# Reactivation of occult hepatitis B virus infection under treatment with abatacept: a case report

**DOI:** 10.1186/s40360-016-0060-2

**Published:** 2016-04-21

**Authors:** Rossella Talotta, Fabiola Atzeni, Piercarlo Sarzi Puttini

**Affiliations:** Rheumatology Unit, University Hospital “Luigi Sacco”, Via GB Grassi 74, 20157 Milan, Italy

**Keywords:** Abatacept, Hepatitis B, Reactivation, Rheumatoid arthritis

## Abstract

**Background:**

Abatacept (ABA) is a fusion receptor protein containing the CTLA-4 domain that prevents the activation of naïve T cells by binding the CD80 and CD86 molecules expressed on the surface of dendritic cells, indicated for the treatment of moderate to severe rheumatoid arthritis (RA). There is still little evidence concerning the safety of ABA in RA patients with positive serology for hepatitis virus B (HBV) infection.

**Case Presentation:**

We report the case of a HBV infection reactivation in an ABA-treated male RA patient. The patient (caucasian race, 66-year-old) was diagnosed with RA in Novembre 2010 and in December 2010 he started a treatment with prednisone plus subcutaneous methotrexate. In October 2011, an anti-TNF agent (golimumab) was added but soon discontinued due to an adverse event. At baseline, screening for HBV markers showed a positivity for HBcAb and HBeAb IgG, being HBsAg, HBsAb, HBcAb IgM, HBeAg and HBV DNA negative. Serum amino-transferase (AST and ALT) levels were within the normal range. In January 2012 he was swapped to intravenous treatment with ABA 750 mg/month, that allowed the achievement of a good clinical response and the permanent discontinuation of corticosteroids. In November 2013, laboratory reports showed that he was positive for HBcAb but negative for the remaining HBV markers, and had a slightly increased AST level and, in December 2013, he became HBV DNA positive (326 IU/mL). In January 2014, his HBV DNA levels had further increased and ABA was stopped while maintaining MTX. He started lamivudine 100 mg/day in January 2014. After 1 month of lamivudine, his HBV DNA levels became undetectable (<10 IU/mL) and liver function was normal although RA had been reactivated (DAS28 5.53). Treatment with ABA was therefore resumed with the achievement of a good response after 6 months. The patient is currently being treated with lamivudine 100 mg/day, i.v. ABA 750 mg/month, and MTX 15 mg/week, with a good response (DAS28 2.27 in October 2015), and constantly monitored without any further evidence of HBV infection reactivation.

**Conclusions:**

Although there are still few reports in literature, we suggest caution in HBV- occult carriers RA patients undergoing a treatment with abatacept.

## Background

The treatment of rheumatoid arthritis (RA) includes many biologic drugs blocking different steps of the inflammatory cascade. Abatacept (ABA) is a fusion receptor protein containing the CTLA-4 domain that prevents the activation of naïve T-cells by binding CD80 and CD86 expressed on the surface of dendritic cells [1]. Abatacept, with or without methotrexate (MTX), is approved for treating moderate to severe RA in patients refractory to previous disease modifying anti-rheumatic drugs (DMARDs) and anti- TNF drugs.

Evidences show the safety of ABA in RA patients with positive serology for hepatitis virus (HBV) infection [2], and there are few reports of HBV reactivation. We observed a case of HBV infection reactivation in a Caucasian male patient affected by RA treated with ABA.

## Case presentation

The patient, 66 years old, was diagnosed with RA in November 2010 according to the new European League Against Rheumatisms (EULAR)/American College of Rheumatology (ACR) 2010 criteria [3]. At baseline clinical examination showed 23 swollen joints and 26 tender joints, increased values of Erythrocyte Sedimentation Rate (ESR), C-Reactive Protein (CRP) and of Rheumatoid Factor (RF) (15 mg/L) and anti-citrullinated peptides antibodies (ACPA). Complete blood count, serum amino-transferases and creatinine were within the range of normality. X-rays of both hands and feet showed juxta articular osteoporosis but no bone erosions.

In December 2010 he started a treatment with prednisone 5 mg/day plus subcutaneous MTX 10 mg/week, which was increased up to 15 mg/week in February 2011. Due to persistent disease activity (DAS28 = 4.16), in October 2011 the patient was considered candidate to a biologic agent according to the Italian guidelines and therefore a screening for opportunistic infections was required [4]. Tuberculosis screening assessed by interferon-gamma release assay (IGRA) proved negative. Thorax X-rays showed no pleural or pulmonary lesion. Hepatitis screening showed a positivity for HBcAb IgG and HBeAb IgG, while HBsAg, HBsAb, HBcAb IgM, HBeAg and HCV-Ab were negative. HBV DNA was negative (<20 IU/mL), AST and ALT value were within the normal range (20 and 27 IU/L respectively). In November 2011 a therapy with golimumab 50 mg every month was started, but it was permanently discontinued after three weeks due to adverse effects (cough and tachycardia). In January 2012 the patient was swapped to a treatment with abatacept (ABA) e.v. 750 mg monthly. At baseline DAS28 was 4.85; CRP value was 19 mg/L (normal values < 1 mg/L), ESR 21 mm (normal values < 27 mm), AST 18 IU/L (normal values < 40 IU/L) and ALT 30 IU/L (normal values < 40 IU/L).

HBV DNA, AST and ALT were evaluated every three months, and remained within the normal range. The response to ABA therapy was good with DAS28 achieving the score of 2.31 after 3 months. The patient maintained a clinical remission at the follow-up visits despite the tapering and the definitive discontinuation of corticosteroids.

However, in November 2013 laboratory reports showed a positivity in HBcAb with a negativity in the remaining HBV serologic markers (HBsAg, HBcAb, HBeAg, HBeAb, HCV-Ab) and a mild increase in the value of AST (41 IU/L). Furthermore, in December 2013 HBV DNA turned positive with a level of 326 IU/mL (normal range <20 IU/mL). In January 2014, a subsequent control of HBV-DNA revealed a further increase up to 1416 IU/mL. ABA was then stopped maintaining MTX, and the patient referred to a hepatologist. A liver examination by means of abdominal ultrasound was done, resulting into normal findings. However, since January 2014 a treatment with lamivudine 100 mg/day was started.

After 1 month of lamivudine, HBV DNA became undetectable (<10 IU/mL) and liver functionality was normal (AST 29 IU/L, ALT 38 IU/L); on the contrary CRP values were greatly increased (42.4 mg/L) and RA reactivated (DAS28 = 5.53); therefore the treatment with ABA was reintroduced. The patient experimented a rapid benefit on the rheumatic symptoms reaching a good EULAR response after 3 months. In April 2014 liver functionality was good (AST 29 IU/L, ALT 31 IU/L), HBV-DNA was <10 IU/mL and an assessment through Fibroscan showed an index of stiffness of 3.9 kPA.

**Fig. 1:**
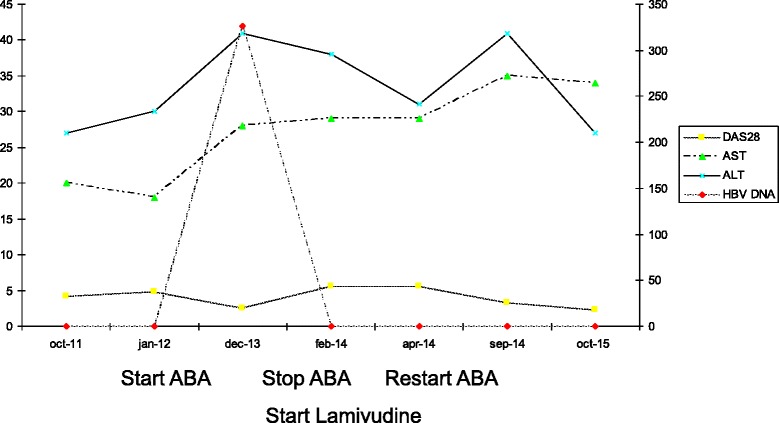
Time-course of DAS28, AST, ALT and HBV DNA levels. DAS28: Disease Activity Score (28 Joints); AST: ASpartate aminotransferase; ALT: ALanine aminotransferase; HBV DNA: Hepatitis B Virus DNA

The patient is currently treated with lamivudine 100 mg/day, i.v. abatacept 750 mg every month and MTX 15 mg/week with a good response (DAS28 = 2.27 in October 2015). Figure 1 illustrates the time-course of DAS28, AST, ALT and HBV DNA levels.

## Conclusions

The association between ABA and HBV infection is still unclear. Germanidis et al. [5] reported the case of a elderly RA woman who developed an active HBV hepatitis after 1 year of treatment with ABA. The patient was concomitantly treated with prednisone 10 mg/day and leflunomide 20 mg/day, perhaps favouring the viral reactivation. Our patient did not need concomitant treatment with prednisone because the RA disease activity was persistently low under ABA treatment (DAS28 < 2.6).

Other authors reported a case of HBV reactivation in an occult carrier receiving ABA as first biologic line for RA [6]. In this case a treatment with tenofovir 300 mg/day was started and ABA was permanently discontinued. A recent “real life” Italian study was carried on 72 RA patients with concomitant HBV infection, treated with ABA plus steroids and conventional Disease Modifying Anti-Rheumatic Drugs (DMARDs). Among them, 21 were occult carriers who received prophylaxis with lamivudine only in 9 cases. No viral reactivation was detected in a 24 months follow-up period, underlining a safe profile of ABA during HBV infection, also without applying antiviral prophylaxis [7]. However, in our experience, a treatment with a biologic agent inducing a peripheral tolerance could favour the progression of HBV infection in an occult carrier. The concomitant use of corticosteroids may represent an aggravating factor, whereas the use of methotrexate may be helpful in preventing the immune-mediated damage to HBV-infected hepatocytes [8]. Therefore, HBV DNA and serum levels of AST and ALT should be periodically monitored in occult carriers. In case of HBV reactivation patients can be successfully treated with antiviral agents while continuing the treatment with ABA [9]. In conclusion, reports remain still isolated in literature and, according to our experience, this is the first case of HBV reactivation under ABA in our cohort of RA patients (n.9) affected by occult HBV infection.

## Consent

Written informed consent for publication of the clinical details was obtained from the patient. A copy of the consent form is available for review by the Editor of this journal.

## Abbreviations

ABA: abatacept
ACPA: anti-citrullinated peptides antibodies
ACR: American College of Rheumatology
CRP: C-reactive protein
DAS28: disease activity score (28 Joint)
DMARDs: disease modifying anti-rheumatic drugs
ESR: erythrocyte sedimentation rate
EULAR: European League Against Rheumatisms
HBV: hepatitis B virus
IGRA: interferon-gamma release assay
MTX: methotrexate
RA: rheumatoid arthritis
RF: rheumatoid factor
